# Tuberculosis Contact Investigation Using Interferon-Gamma Release Assay with Chest X-Ray and Computed Tomography

**DOI:** 10.1371/journal.pone.0085612

**Published:** 2014-01-14

**Authors:** Akira Fujikawa, Tatsuya Fujii, Satoshi Mimura, Ryota Takahashi, Masao Sakai, Shinya Suzuki, Yukishige Kyoto, Yasuhide Uwabe, Shinji Maeda, Toru Mori

**Affiliations:** 1 Department of Medical Education and Research, Japan Self-Defense Forces Central Hospital, Ikejiri, Setagaya, Tokyo, Japan; 2 Department of Radiology, Japan Self-Defense Forces Central Hospital, Tokyo, Japan; 3 Department of Internal Medicine, Kawakita General Hospital, Asagaya-kita, Suginami, Tokyo, Japan; 4 Division of Respiratory Medicine, Department of Internal Medicine, Japan Self-Defense Forces Central Hospital, Tokyo, Japan; 5 Division of Infectious disease, Department of Internal Medicine, Japan Self-Defense Forces Central Hospital, Tokyo, Japan; 6 Department of Internal Medicine, Japan Ground Self-Defense Force Fukuoka Hospital, Kokura-Higashi, Kasuga, Fukuoka, Japan; 7 Molecular Epidemiology Division, Department of Mycobacterium Reference and Research, Research Institute of Tuberculosis Japan, Matsuyama, Kiyose, Tokyo, Japan; 8 Japan Anti-Tuberculosis Association (JATA), Research Institute of Tuberculosis Japan, Matsuyama, Kiyose, Tokyo, Japan; Institute of Infectious Diseases and Molecular Medicine, South Africa

## Abstract

Between September 2009 and January 2010, 6 members of the Japanese Eastern Army, who had completed the same training program, were diagnosed with active tuberculosis (TB) on different occasions. The Ministry of Defense conducted a contact investigation of all members who had come into contact with the infected members. The purpose of this study was to verify the efficacy of the TB screening protocol used in this investigation. A total of 884 subjects underwent interferon-gamma release assay (IGRA) and chest X-ray. The 132 subjects who were IGRA positive or with X-ray findings suggestive of TB subsequently underwent chest computer tomography (CT). Chest CT was performed for 132 subjects. Based on CT findings, 24 (2.7%) subjects were classified into the active TB group, 107 (12.1%) into the latent tuberculosis infection (LTBI) group, and 753 (85.2%) into the non-TB group. The first 2 groups underwent anti-TB therapy, and all 3 groups were followed for 2 years after treatment. Although one subject in the active TB group experienced relapse during the follow-up period, no patient in the LTBI or non-TB groups developed TB. IGRA and chest X-ray, followed by chest CT for those IGRA positive or with suspicious X-ray findings, appears to be an effective means of TB contact screening and infection prevention.

## Introduction

As a country with an intermediate tuberculosis (TB) burden, Japan occasionally reports an outbreak or case of group infection of TB. Between September 2009 and January 2010, 6 cases of active TB were reported within the jurisdiction of the Eastern Army, Ground Self-Defense Force (SDF). An epidemiological study revealed that all 6 members had completed a training program for new staff members that had been held at the same military post between April and June 2009. To prevent the further spread of TB according to Article 17 of the Japanese Law of Infectious Disease, the Medical Department of the Ground Staff Office, Ministry of Defense, decided to conduct temporary health check-ups as a rapid means of screening surveillance for those who had been exposed to the infected members.

Prior contact TB screening consisted of health check-ups, an interview, chest X-ray, and tuberculin skin test (TST). TST results were obtained by measuring skin redness and the presence or absence of cutaneous induration 48 hours and 8 weeks after intradermal injection of purified protein derivative, using results from a previous TST performed at the time of military enrollment for reference. Increased size of skin redness or presence of cutaneous induration was defined as positive TST. Subjects with a positive TST and suspicious CXR findings were treated as active TB patients. Subjects with positive TST and negative CXR findings were considered to represent latent tuberculosis infection (LTBI) and received preventive TB treatment, depending on their proximity to an index case or clinical symptoms and signs.

In conducting screening for group infection, completing the task as rapidly and accurately as possible is important to preventing the spread of infection and emergence of an epidemic. A major challenge in completing this task has been, and continues to be, the lack of an established screening protocol for contact investigation of TB group infection appropriate for the conditions faced by the Ground SDF members, who are highly mobile and often act in groups over a broad area extending beyond several local municipalities. According to Article 17 of the Japanese Law of Infectious Disease, TB contact screening has been performed in each military post in collaboration with local government; however, the intensification of the management and information-gathering of the disease by the Japan Ground SDF and the SDF Central Hospital has appeared to be insufficient. Furthermore, when the protocol for this contact investigation was created by the Health Management Center of the Japan SDF Central Hospital, several limitations were expected regarding screening using the traditional TST. First, uncertainty was expected regarding the reliability of the test results because all the subjects had previously received the Bacillus Calmette–Guerin (BCG) vaccination. Second, confusion regarding the results was expected due to inconsistent reading of the TST across military posts, as the participants had been assigned to 35 different military work posts after completing training. Third, much time was expected to be necessary to complete the screening process, and repeated visits by the examinees were anticipated.

In consideration of these limitations, this contact investigation conducted screening for tuberculosis using interferon-gamma release assay (IGRA) rather than the TST. IGRA uses specific antigens of *Mycobacterium tuberculosis,* such as early antigenic target-6 (ESAT-6), culture filtrate protein 10 (CFP-10), and TB 7.7, to stimulate whole blood to induce interferon-gamma production by antigen-specific T cells. Therefore, the IGRA results are not affected by history of BCG vaccination. They are presented in terms of numerical values, allowing for objective interpretation, and are produced within a relatively shorter time frame. Furthermore, studies of contact investigations of TB group infection that used the IGRA and TST have reported that the detection accuracy of the IGRA is equal to or better than that of the TST [1]–[6]. However, IGRA alone cannot distinguish between active TB and LTBI, which is important for differential treatment of these conditions [7], [8]. For this reason, chest computed tomography (CT) was also used to detect possible early pulmonary TB, which may not be detected by chest X-ray [9], in the protocol.

Here, we verified the efficacy of initial screening consisting of IGRA prior to chest X-ray and chest CT for the management of TB group infection in a highly mobile population that often acted in groups.

## Methods

**Figure 1:**
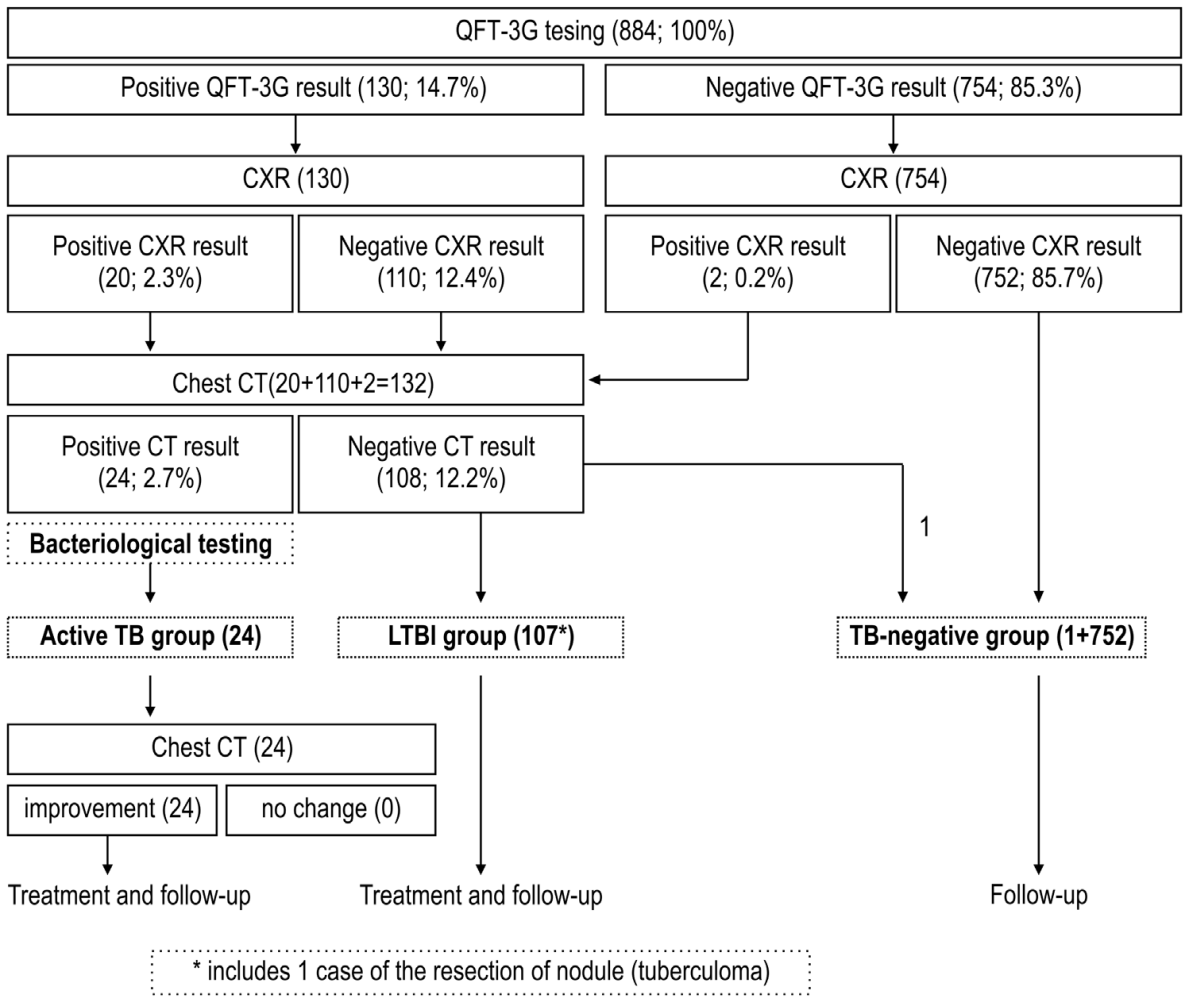
Contact investigation protocol for tuberculosis. The subjects were stratified into 3 groups according to the results of QFT-3G, chest X-ray, and chest CT.

The protocol for this contact investigation was approved by the Medical Department of the Ground Staff Office, Ministry of Defense, and the Research Review Committee of the Japan SDF Central Hospital, and was implemented at the initiative of the Army Surgeon, Eastern Army. Written consent was obtained from all subjects. Contact investigation of 884 subjects, including those who had completed training for new staff members and other staff (mean age, 23.4±5.7 years; range, 19–53 years) and excluding 86 people who had resigned their commission before the initiation of the study, began in March 2010. Collection of blood samples for IGRA and performance of medical examinations and interviews at the 35 military posts at which the subjects had been assigned also began in March 2010. Blood samples were sent to Japan SDF Central Hospital for evaluation. Chest X-rays were obtained for all subjects in April 2010. For those found either IGRA positive or IGRA negative but with chest X-ray results suggesting pulmonary TB, chest CT was performed between April and June 2010 (Fig. 1).

### (1) Interferon-gamma Release Assay

For IGRA, the QuantiFERON–TB Gold in Tube test (QFT-3G; Cellestis, Carnegie, Australia) was used. Three milliliters of whole blood was drawn from each subject and treated according to the manufacturer’s protocol. A QFT-3G test result of less than 0.35 IU/ml was considered negative for TB. Repeated QFT-3G testing of subjects found to have active TB or LTBI was conducted from 1 year after initial diagnosis.

### (2) Chest X-ray Examination

A digital radiographic system (RADREX-iDRAD-3000A; Toshiba, Tokyo, Japan) was used to obtain chest X-rays in the posterior–anterior direction. Diagnosis of possible pulmonary TB was made independently using a 3-megapixel monochrome LCD monitor (ME315L plus; Totoku Electric, Tokyo, Japan) by one radiologist with 23 years of experience and 2 respiratory medicine physicians with 34 and 16 years of experience, respectively, who were blind to the QFT-3G results. Each reader read a subset of images and independently classified the result as positive or negative. CXR findings considered suspicious for active TB include infiltration, atelectasis, cavitation, clusters of nodular opacities, diffuse nodular opacities, and pleural effusion. If any findings were suggestive of active pulmonary TB, the subject was categorized as chest X-ray positive.

### (3) Chest CT Examination

Chest CT was performed using a 6-row multi-detector row CT machine (SOMATOM Emotion 6; Siemens, Tokyo, Japan) set at the parameters typically used at the facility (130 kVp, effective current 95 mAs, 2-mm collimation × 6, pitch of 1.4). Based on measurements of the dosimetry phantom (diameter 32 cm, length 35 cm) under automatic exposure control by dose-modulation software (Software CARE Dose4D; Siemens, Tokyo, Japan), the radiation exposure of each subject was estimated to be less than 2.8 mSv. Two types of axial images (5-mm thickness/5-mm interval and 2.5-mm thickness/1.5-mm interval, respectively) were obtained and reconstructed before being reviewed on the picture archiving and communication system monitor by 2 radiologists with 23 and 19 years of experience, respectively, who were blind to the chest X-ray results. Positive CT criteria were based on the report by Im et al. [10]. A positive chest CT result was indicated if one or more of the following evaluation criteria was observed: 1) consolidation, 2) cavitation, 3) clusters of non-calcified nodules ≤4 mm in diameter associated with dilated or thickened peripheral airway walls, 4) non-calcified nodules >4 mm in diameter with adjacent small nodules, and/or 5) widespread distribution of small nodules 1–4 mm in diameter. The presence or absence of lymphadenopathy or pleural effusion was also recorded.

### (4) Bacteriological Testing

Depending on whether the subject was able to expectorate sputum, a respiratory or gastric fluid sample was collected at least 3 times from all subjects who were chest-CT positive, regardless of QFT-3G status. In addition to acid-fast smear testing, mycobacterial culturing was performed using solid 1% Ogawa egg medium (Nissui, Tokyo, Japan), incubated in tubes at 37°C and observed weekly for up to 8 weeks. In accordance with the manufacturer’s protocol, observation of more than one colony was considered a positive result. Subsequent genetic analysis of cultured *M. tuberculosis* was performed using the restriction fragment length polymorphism (RFLP) technique, which consists of agarose gel electrophoresis, Southern blotting, and hybridization after fragmentation of an extracted DNA sample by restriction enzyme to determine the length of the resulting DNA fragments.

### (5) Diagnostic Criteria for Active TB and LTBI Groups

After the possibility of community-acquired pneumonia had been excluded, subjects with positive chest-CT results either with or without positive bacteriology were diagnosed with active TB and began a treatment regimen for active tuberculosis consisting of 300 mg isoniazid, 450 mg rifampicin, 750 mg ethambutol, and 1000 mg pyrazinamide per day for 26 weeks. Chest CT examination was performed 6 months after treatment initiation to determine the extent of improvement.

Subjects with positive QFT-3G results and negative chest CT/bacteriological results were diagnosed with LTBI and, after receiving information regarding the Directly Observed Treatment, Short Course, began a regimen of anti-TB drugs, either 300 mg isoniazid or 450 mg rifampicin per day, for 26 weeks. In the 2 years following completion of treatment, subjects underwent a follow-up examination every 6 months consisting of medical examination, an interview, and chest X-ray, as well as sputum bacteriological examination if any change on chest X-ray was observed.

### (6) Comparison of QFT-3G Values

The Mann–Whitney U-test was used to compare the QFT-3G values obtained for the active TB and LTBI groups at screening and the values obtained for the *M. tuberculosis* culture-positive and culture-negative subgroups of the active tuberculosis group at screening. The Wilcoxon signed-rank test was used to compare the values obtained for the active TB and LTBI groups at screening and after treatment if QFT-3G values had been obtained during the follow-up period. Excel 2010 software (Microsoft, Tokyo, Japan) was used for all statistical analyses.

## Results

**Figure 2:**
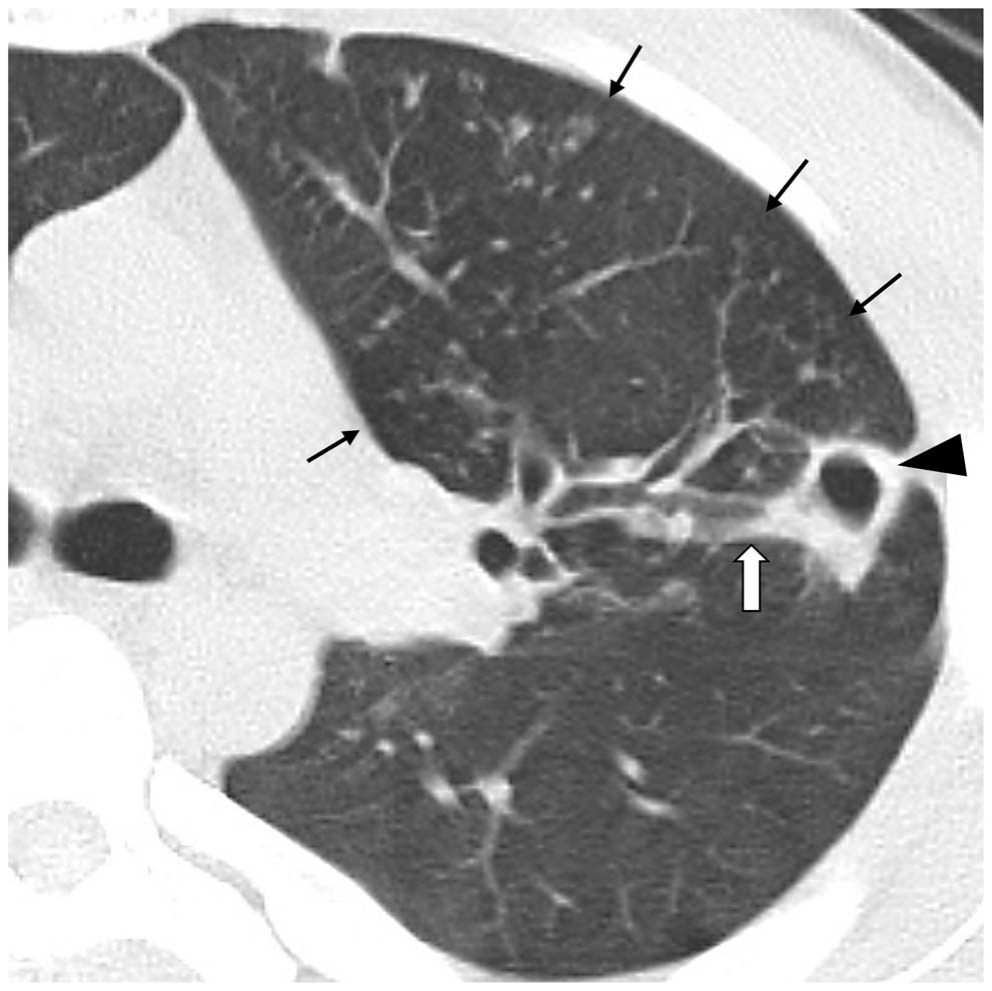
CT findings of active TB. A chest CT image of the left upper lobe of the lung in a 19-year-old subject demonstrated clustered non-calcified nodules associated with dilated peripheral airways (thin arrows) and a cavitation (arrow head) with dilated and thickened subsegmental bronchus (white arrow).

Of the 884 subjects, 130 (14.7%) were QFT-3G positive, and 754 (85.3%) were QFT-3G negative. Of the 22 subjects with chest X-ray images suggestive of TB, 2 were QFT-3G negative and subsequently underwent chest CT. Of the 110 subjects who were QFT-3G positive and chest X-ray negative, 8 subsequently underwent chest CT examination at other facilities because of their remote work locations. Review of the chest CT of the 132 subjects, including 130 QFT-3G-positive subjects and 2 QFT-3G-negative subjects with positive chest X-ray, indicated that 24 were positive and 108 negative for active TB. Review of the chest CT results of the 2 subjects with negative QFT-3G results indicated that one was positive and one was negative for active TB (Fig. 1). All 24 subjects with positive chest CT examination results demonstrated clusters of non-calcified nodules associated with abnormal peripheral bronchioles. While 15 also had non-calcified nodules with adjacent small nodules and 3 also had cavitation, none demonstrated lymphadenopathy or pleural effusion (Fig. 2). No risk factors such as HIV infection or diabetes were noted among the 24 active TB subjects. Twenty of the 24 subjects in the active TB group and 39 of 108 subjects in the LTBI group had resided on the same floor of the barrack. At the time of screening, no significant clinical symptoms or signs were noted, although almost all subjects recalled a passing mild cough similar to a common cold during the training period.

### (1) Active TB Group

**Figure 3:**
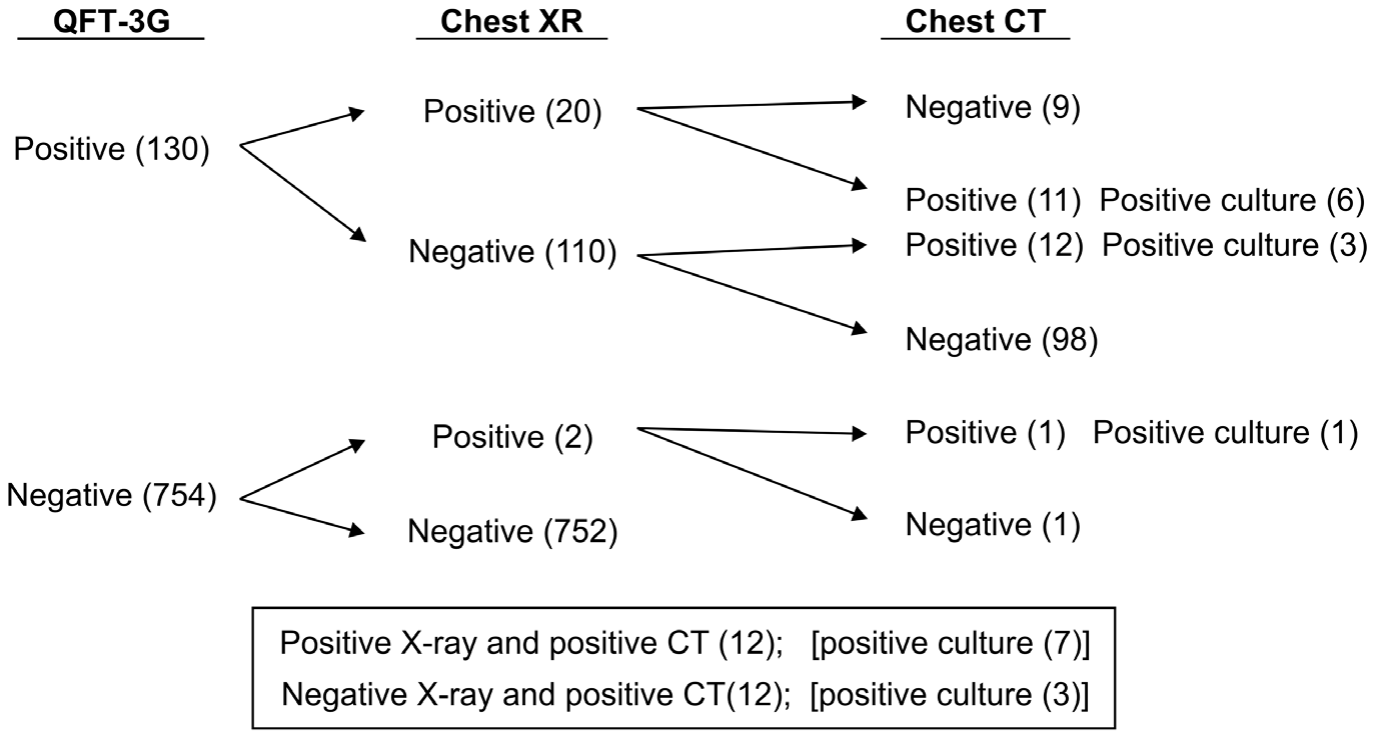
Bacteriologically positive cases of active tuberculosis diagnosed by the investigation protocol. Ten of 24 subjects diagnosed with active TB in the protocol showed positive culture for *M. tuberculosis*. Of these 10 subjects, 3 were missed by chest X-ray.

In this investigation, 12 of 24 active TB subjects were chest X-ray negative, while 9 of 20 subjects who were both QFT-3G and chest X-ray positive, were chest CT negative (Fig. 3). Therefore, 11 of the 23 active TB subjects with positive QFT-3G were both chest X-ray and chest CT positive.

Culture testing of either a sputum or gastric fluid sample from each subject indicated that 10 of the 24 subjects who had been diagnosed with active TB were culture-positive for *M. tuberculosis*. Subsequent RFLP confirmed that the cultured *M. tuberculosis* samples from these 10 subjects were of an identical genotype. The genotype was also identical to that obtained from one of the original 6 active TB cases. Review of the chest X-ray findings of the 10 subjects indicated active TB in 7, of whom one was QFT-3G negative (Fig. 3). The signs of TB in the CT findings of all subjects were found to have either improved or disappeared at the second chest CT examination performed 6 months after treatment initiation. However, one subject who experienced relapse of pulmonary TB 8 months after completion of the initial treatment was required to resume anti-tuberculosis treatment.

### (2) LTBI Group

Of the 107 subjects diagnosed with LTBI, 4 who suffered adverse effects from isoniazid were subsequently treated with rifampicin. No TB development was observed in any of the subjects during the 2-year follow-up period after completion of anti-TB treatment. Of the 108 subjects with negative chest CT results, 10 had abnormal findings that did not meet the criteria of active TB: 4 had thick linear attenuation; 2 had solitary small ground-glass attenuation; and 1 each had arteriovenous malformation, pulmonary cyst, solitary calcified nodule, and solitary nodule. When, upon the subject’s request for further evaluation, surgical resection of the lesion of one subject with a solitary nodule was performed, a tuberculoma was detected. Subsequent chest CT examination prior to anti-TB drug administration revealed that the ground-glass attenuation that had been observed in 2 of the subjects had disappeared.

### (3) Non-TB Group

No TB development was observed in the non-TB group during the 2-year follow-up period.

### (4) Comparison of the QFT-3G Values

**Figure 4:**
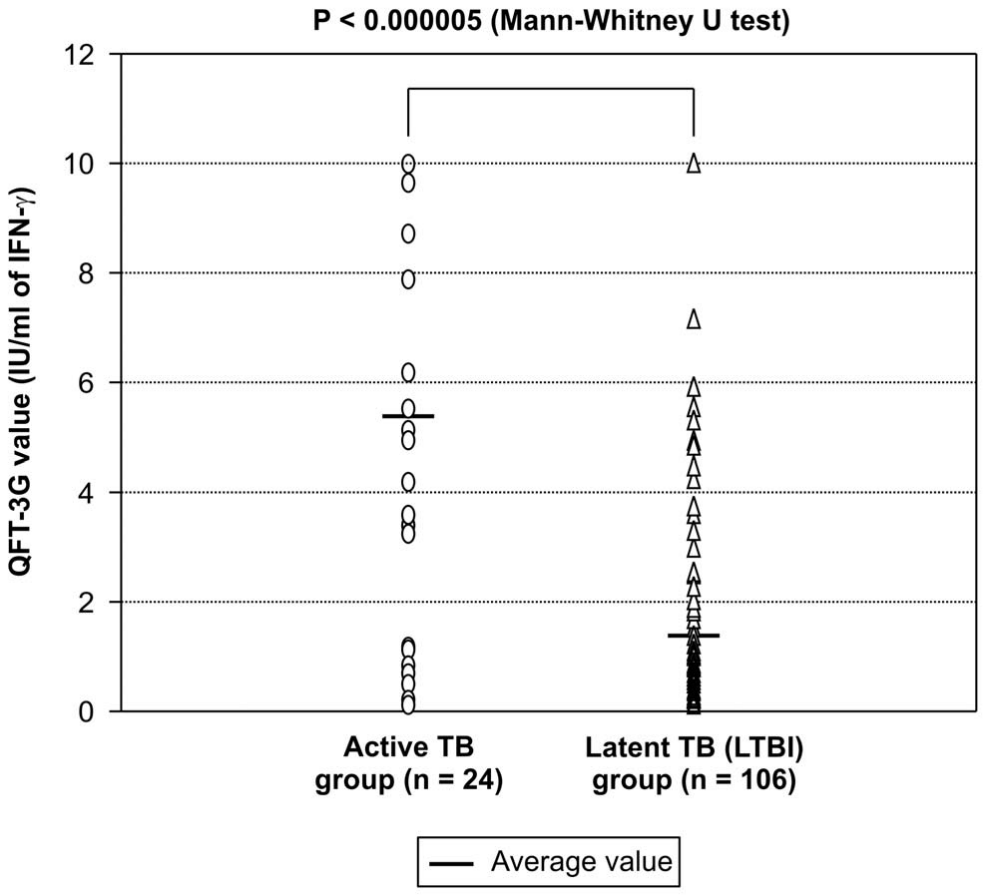
Comparison of QFT-3G values at screening between the active TB group and latent (LTBI) group. Comparison showed a statistically significantly higher value in the active TB group than in the LTBI group.

**Figure 5:**
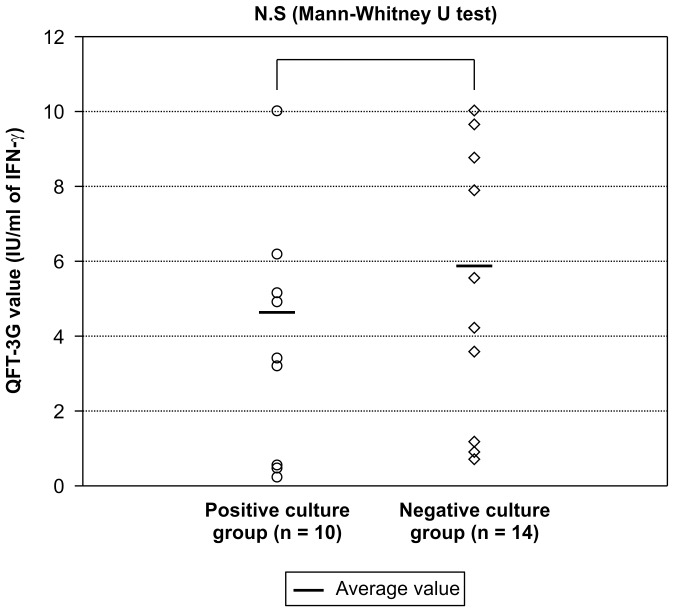
Comparison of QFT-3G values between the positive and negative culture groups at diagnosis of active TB. Comparison showed no significant difference between the 2 groups.

**Figure 6:**
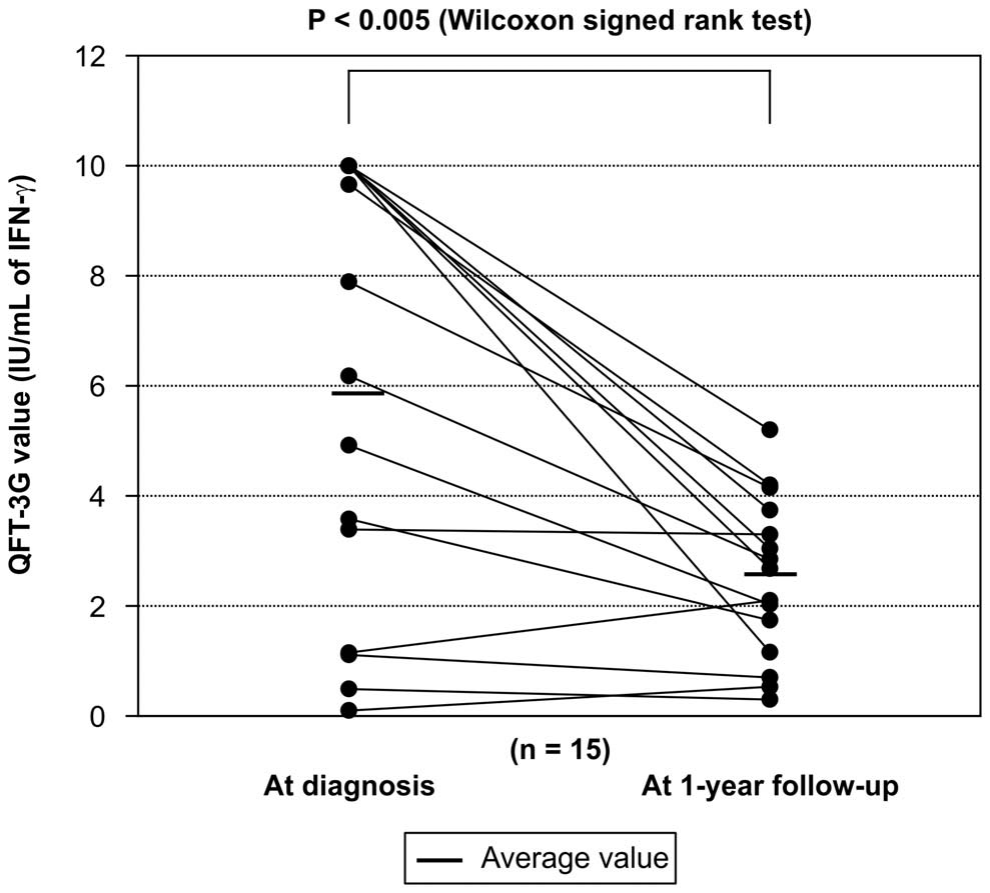
Comparison of QFT-3G values at diagnosis and after treatment of active TB. Comparison showed a statistically significant reduction of QFT-3G values after treatment.

**Figure 7:**
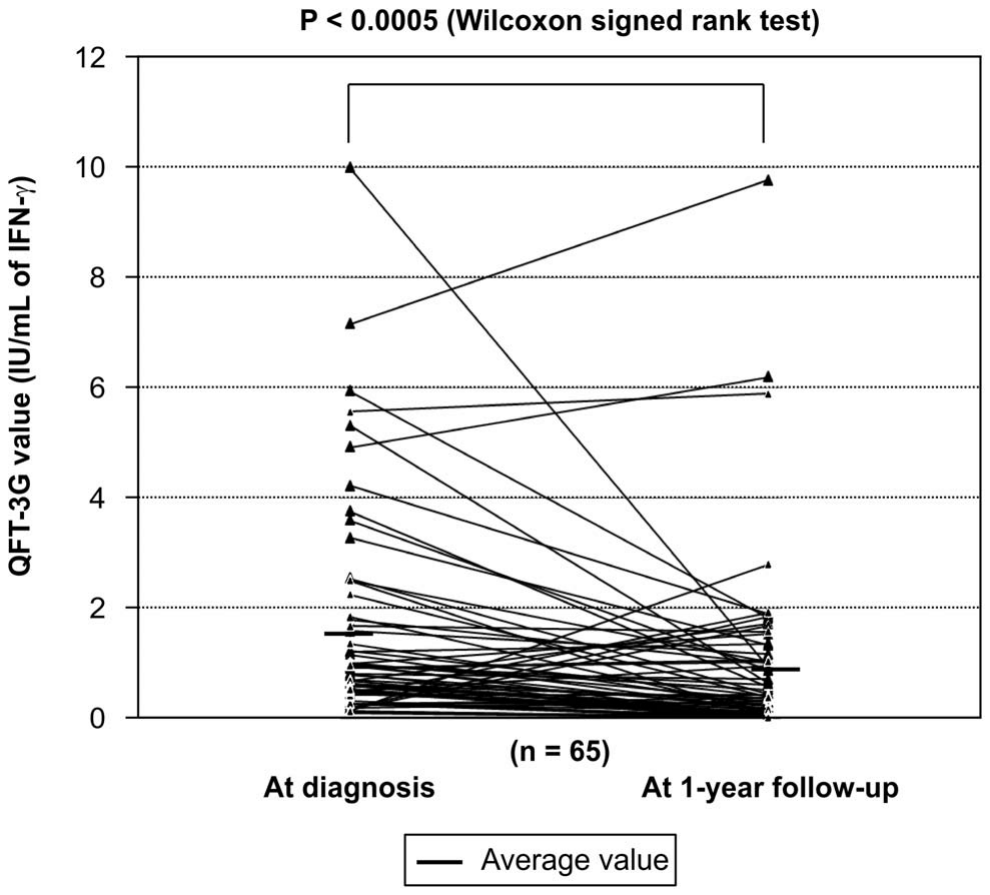
Comparison of QFT-3G values at diagnosis and after treatment of latent TB. Comparison showed a statistically significant reduction of the QFT-3G values after treatment.

Repeated QFT-3G testing of 15 subjects in the active TB group and 65 subjects in the LTBI group was performed 1 year after initial diagnosis. Due to the limitations of the sampling date schedule for QFT-3G in our outpatient clinic, subjects’ training schedule, and remote work locations, repeated QFT-3G testing could not be performed for all subjects in the active TB and LTBI groups. Comparison of the QFT-3G values of the active TB and LTBI groups at screening indicated that the values of the former were statistically significantly higher than those of the latter (Fig. 4). Comparison of the QFT-3G values for the *M. tuberculosis* positive- and negative-culture subgroups of the active TB group indicated no significant differences between the 2 groups (Fig. 5). Comparison of the QFT-3G values obtained at screening and follow-up indicated that the values for both the active TB and LTBI groups had significantly decreased at follow-up (Fig. 6 and 7).

### (5) Determination of the Sensitivity and Specificity of the QFT-3G Test

Because it was uncertain whether the case with tuberculoma had any correlation with this group infection, the case was excluded from this analysis. The sensitivity of the QFT-3G screening test in detecting active tuberculosis was found to be 95.8% (23/24), and the specificity was 87.7% (753/859).

## Discussion

As no signs of new infection were detected in either the LTBI or TB-negative groups during the 2-year follow-up period, the contact surveillance conducted in this study, which was composed of the complementary screening methods of QFT-3G testing, chest X-ray, and chest CT, appears to have achieved effective prevention of infection spread.

The QFT-3G-positive rate of the subjects in this study was 14.7%, which, being much higher than the estimated rate of approximately 1% in the young Japanese adult population, indicates that the subjects had likely experienced very close group contact. We acknowledge the possibility of some of the active TB subjects diagnosed in this investigation, might have had a prior contact [11].

IGRA alone cannot be used to distinguish between active TB and LTBI. Previous contact investigations have reported the rate of IGRA-positive subjects who develop active TB to range from 10% to 30% [12], [13]. In the current study, subjects found to be QFT-3G positive but chest-CT negative were diagnosed with and treated for LTBI, although the only gold standard for diagnosis of LTBI is development of active TB [8]. As the population studied here is very mobile and maintains close contact within groups, treating LTBI members is important for group infection management, leading to an inevitable tendency toward overdiagnosis. Interestingly, our findings that QFT-3G values were higher in the active TB group than in the LTBI group and that these values decreased after treatment are consistent with previous studies investigating the quantitative discrimination between active TB and LTBI [14]–[16] (Fig. 4, 6, 7). However, because all subjects diagnosed with LTBI were treated, we were unable to establish a cut-off value by which to determine the necessity of preventive therapy for subjects with positive QFT-3G test results but negative chest CT results.

The definitive gold standard for diagnosing active TB is positive *M. tuberculosis* culture of a sputum or gastric fluid sample. A major challenge in TB testing has been underdiagnosis due to factors such as test technique, test accuracy, or patient disease state [17], [18]. Of the 24 subjects diagnosed with active TB, 10 tested positive for *M. tuberculosis*, yielding a culture-positive rate (41.7%) similar to that reported by Lee et al. [19]. The early active TB cases detected using chest CT may have been culture negative because early active TB is believed to be relatively more common in contact investigations. The lack of overt symptoms of TB also can be suggestive of early infection. Furthermore, no significant differences between the QFT-3G values obtained for the culture-positive and culture-negative groups were observed among subjects who had been definitively diagnosed with active TB based on CT findings (Fig. 5). Nonetheless, we acknowledge the possibility that not all subjects in the active TB group would necessarily progress to severe disease, which should be aggressively treated. However, we emphasized control of the disease in our target population.

One advantage of chest CT may be the prevention of development of isoniazid-resistant *M. tuberculosis*, a risk posed by the use of isoniazid therapy alone for active TB subjects who were misdiagnosed with LTBI by positive QFT-3G and negative X-ray results. Another advantage of chest CT is the identification of false-positive chest X-ray results.

Although it is a very effective tool in group infection management, CT screening poses the risk of excessive radiation exposure. Use of dose-modulation software has been estimated to achieve an approximate 25% reduction in the effective dose of 2.8 mSv per chest CT examination [20]. Considering that the average natural background radiation level in the US is 3.1 mSv, the positive benefits of chest CT appear to outweigh its risks. Moreover, the cost-effectiveness of the methods used in this contact investigation have been verified by a recent study reporting that a combined QFT-3G-testing and CT-examination strategy yielded the combined greatest benefit at the lowest cost of all possible combinations of TB screening tests [21].

Several aspects of this study may limit the generalization of the findings. One limitation is the study sample, which was composed of healthy adults approximately 20 years of age and among whom the percentage found to be QFT-3G negative and chest X-ray negative was relatively low. Older populations are expected to have higher positive rates of QFT-3G or chest X-ray findings in countries with an intermediate or high TB burden. Thus, it is possible that the need for chest CT examination to detect active TB increases with age. A second limitation is the inability to follow subjects who had resigned their commission before the initiation of the investigation. A third limitation is that results obtained using IGRA and TST were not compared. However, considering the characteristics of the target population, such as history of BCG vaccination in all subjects and time and personnel constraints, IGRA can be considered useful.

In conclusion, the screening methodology of TB contact investigation used in this unique setting for a highly mobile population, after very close group contact, was appropriate and effective. Specifically, use of a combination screening protocol of QFT-3G testing and chest X-ray examination appeared to be effective in ruling out infection in subjects with no signs of TB, while a protocol of QFT-3G testing and chest CT examination also appeared to be effective in detecting active TB and, thus, differentiating between cases of active and latent TB.
